# Predictors of mental well-being over the first lockdown period due to the COVID-19 pandemic in France. A repeated cross-sectional study

**DOI:** 10.3389/fpubh.2023.1234023

**Published:** 2023-08-28

**Authors:** Guillaume Barbalat, Audrey Tanguy Melac, Elodie Zante, Frédéric Haesebaert, Nicolas Franck

**Affiliations:** ^1^Centre Ressource de Réhabilitation Psychosociale, Centre hospitalier Le Vinatier, Bron, France; ^2^UMR 5229, CNRS and Université Claude Bernard Lyon 1, Université de Lyon, Lyon, France; ^3^Pôle Centre rive gauche, Centre hospitalier Le Vinatier, Bron, France; ^4^Health Data Department, Lyon University Hospital, Lyon, France; ^5^PSYR2, INSERM U1028, CNRS UMR 5292, CRNL, Université de Lyon, UCBL, Lyon, France

**Keywords:** COVID-19, mental health, well-being, lockdown, public health

## Abstract

**Introduction:**

Numerous studies have investigated the positive and negative effects of potential predictors of well-being during lockdowns due to COVID-19. Yet, little is known on whether these effects significantly changed with time spent in lockdown. In the current study, we described the association of mental well-being with a large number of background characteristics (e.g., socio-demographic or health-related factors), COVID-related factors, and coping strategies, over the duration of the first lockdown due to COVID-19 in France.

**Methods:**

A nationwide online survey was conducted over 7 of the 8 weeks of the 1st lockdown in France, i.e., from 25 March 2020 to 10 May 2020. The level of mental well-being was reported using the Warwick-Edinburgh Mental Well-Being Scale (WEMWBS). We also measured various background characteristics (e.g., age, sex, education, health issues), COVID-related factors (e.g., health and economic risks, agreement with lockdown), and coping strategies. Our analytical strategy enabled us to disentangle effects aggregated over the study period from those that linearly vary with time spent in lockdown.

**Results:**

Our final dataset included 18,957 participants. The level of mental well-being dropped gradually from the third to the eighth week of lockdown [49.7 (sd 7.9) to 45.5 (sd 10.6)]. Time in lockdown was associated with a decrease in well-being (for each additional 10 days of lockdown: B = −0.30, 95%CI: −0.62, −0.15). Factors that showed significantly negative and positive effects on well-being as time in lockdown progressed were (for each additional 10 days of lockdown): having current psychiatric problems (B = −0.37; 95%CI: −0.63, −0.04), worries about having access to personal protective equipment (B = −0.09; 95%CI: −0.18, −0.01), coping by having positive beliefs about the future of the pandemics (B = 0.29; 95%CI: 0.04, 0.62), being supported by neighbors (B = 0.24; 95%CI: 0.04, 0.44), and being involved in collective actions (B = 0.23; 95%CI: 0.04, 0.46).

**Discussion:**

Participants from our sample saw a drop in their mental well-being throughout the first period of COVID-19 lockdown. Policymakers should be mindful of factors contributing to greater deterioration of mental well-being over time, such as having current psychiatric issues. Promoting collective actions and local support from neighbors may alleviate the deterioration of mental well-being over time.

## 1. Introduction

When the World Health Organization (WHO) declared the coronavirus disease 2019 (COVID-19) outbreak a pandemic on 11 March 2020, severe lockdown restrictions were implemented by the governments of several countries worldwide to prevent the uncontrolled spread of the virus. The French government introduced strict social distancing rules on 16 March 2020. Like many other countries all over the world, this was the first time the population of France had ever been subjected to a lockdown to minimize the spread of infectious disease. For over 2 months, under stay-at-home orders, people’s home became the only places where they could sleep, eat, work, do sport (within a 1 km perimeter), and socialize.

The fear of the virus triggered in the minds of virtually everyone who heard about it. Yet, beyond the epidemic itself, measures used to control the spread of the virus and ensuing lifestyle disruptions, uncertainty and loss of control, have also been shown to have a negative impact on mental health (1–4). A number of mental health-related outcomes deteriorated during lockdown, such as affective symptoms (e.g., depressive and anxiety symptoms), quality and quantity of sleep, or quality of life (5). As a result, the use of at least one psychotropic drug increased by 20% compared to pre-lockdown (5). In France, the early phase of COVID-19 containment led to widespread increases in addiction-related habits in the surveyed populations (6), increased anxiety (7) and decreased well-being (8).

The WHO describes mental well-being as a state in which an individual realizes his or her own abilities, can cope with the normal stresses of life, can work productively and fruitfully, and is able to make a contribution to his or her community (9). Well-being is usually conceptualized with two main components: subjective or hedonic well-being on the one hand, and eudaimonic or psychological well-being on the other hand (10). Hedonic well-being includes life satisfaction, positive emotions, and a low level of negative emotions. Eudaimonic well-being may be described as less subjective, and more related to psychological functioning, including dimensions such as self-acceptance, personal growth, purpose in life, positive relations with others, environmental mastery, autonomy (11). While hedonic well-being is usually associated with “feeling good,” eudaimonic well-being is associated with “functioning well” (12). Overall, both these dimensions have been shown to be highly correlated with one-another, depending on a single underlying factor (13).

The level of an individual’s well-being depends on a number of elements, such as demographic characteristics (age, sex, marital status), social factors (socio-economic status, having children, social contacts), personality traits, life events, health, and activities (14). For the purpose of this study, we chose to classify factors influencing well-being during COVID in three different categories: background characteristics (socio-demographic and health-related factors), COVID-related factors (and especially distress linked to COVID policies or related economic consequences), and coping strategies.

First, background characteristics have been related to well-being during COVID-19 lockdown. For instance, female gender (5, 15–17), young age (18, 19), not being in a relationship (20, 21), being a smoker (5), living in a small place, without outdoor space or in an urban area (22–26), or less intuitively having longer periods of physical activity before lockdown (5), have been considered as significant risk factors of decreased well-being during lockdowns. Similar effects were found for students (27), those with low educational attainment (20) and those who are unemployed (28, 29). The effect of lockdown and social distancing on mental health in individuals with pre-existing medical or mental illness has also been demonstrated (17, 30, 31).

Second, well-being may be impacted by a range of factors related to the pandemic itself. The risk of being exposed to the virus is a threat to mental health (32), but perhaps not as highly as expected (33, 34). Other COVID-related factors may be at stake such as a lower trust in, as well as lack of agreement or satisfaction with governmental policies to curb the spread of the virus (35–37). Finally, the pandemic has led to a range of economic consequences that may also increase one’s level of stress, such as financial loss (33), scarcity of essential products (32) and personal protective equipment (PPE).

Third, how one copes with the occupational difficulties associated with the pandemic is also of crucial importance to maintain an acceptable level of well-being (32, 38–40). Examples of coping strategies that have been demonstrated to protect against the negative effects of lockdown are: resilience (41), positive thinking, and seeking social support (42, 43).

Previous research has also investigated whether different subgroups were associated with various effects on mental well-being depending on the temporal course of lockdowns (44–50). These effects may reflect growing fear of catching the virus and/or increased impact of having to maintain lockdown measures. These temporal changes are important to consider as they may disproportionately affect specific population subgroups that need to be identified and supported in order to anticipate and prevent further deterioration of mental health as well as later psychiatric or physical disorders (51, 52). A deterioration of well-being over time may also contribute to increased hopelessness and de-motivation to follow recommended protective behaviors (53).

Studies mentioned above have identified such factors as female gender, young age, past mental issues, social disadvantage, and certain psychological traits, to be related to increased deterioration of mental well-being with time spent in lockdown. Yet, to our knowledge, none of these studies has investigated the temporal effects of a broad variety of predictors in the same model, which would enable the estimation of the importance of each predictor above and beyond all the others.

In the current study, we aimed to determine how the above mentioned factors (background characteristics, COVID-related factors, coping strategies) influenced well-being over the 2 months period of the first COVID-19 lockdown in an adult population in France. Our rationale for this study was twofold. First, we wished to confirm or infirm previous findings with a large sample of French individuals. Second, none of these studies has investigated the temporal effects of a broad variety of predictors in the same model, which would enable the estimation of the importance of each predictor above and beyond all the others.

We used responses to an anonymous repeated cross-sectional online survey from March 25 to May 10 2020. Previous reports have been published using this survey (6, 8) but focused on the initial lockdown period. Here, our analytical strategy enabled us to disentangle overall main effects aggregated over the study period from those that linearly vary with time spent in lockdown. We hypothesized that factors that have been found as significant predictors of mental health outcomes in previous studies would be associated with decreased well-being during COVID-19 lockdown (e.g., female gender, young age/being a student, not being in a relationship, having mental issues, worrying about financial precarity or not having access to essential products, lack of coping strategies).

## 2. Materials and methods

### 2.1. Data

#### 2.1.1. Data source

A nationwide online survey (LockUwell) was launched during the second week of the first lockdown period in France and continued until the end of this period, i.e., till the end of the 8^th^ week of lockdown. The data were collected from 25 March 2020 to 10 May 2020. No cohort was involved: different participants responded at different time points. The methodology and reporting of the results are based on the Checklist for Reporting Results of Internet E-Surveys (CHERRIES) (54).

The survey was specially developed for this study and it has already been described by Haesebaert et al. (8). Briefly, participants were recruited via online announcements on social networks, the websites of national newspapers, and mailing lists using a convenience non-sampling method, with no incentives. The questions aimed to collect a range of items relating to mental well-being and to embrace a large range of socio-demographic and environmental data relating to the lockdown situation. The questionnaire was divided into six sections: socio-demographic data (section 1), assessment of well-being over the week preceding the response date (French version of the Warwick-Edinburgh Mental Well-being Scale, WEMWBS) (13, 55) (section 2), Visual Numerical Scales for stress (section 3), history (section 4), personal situation regarding COVID-19 (i.e., whether respondents had or knew someone who had COVID-19 and personal feelings regarding COVID-19) (section 5), as well as personal and environmental conditions during lockdown (section 6). We extracted data from all the above-mentioned sections except section 3, as only well-being (and not stress) was the focus of this study.

#### 2.1.2. Outcome variable

The WEMWBS is a 14-item measure of mental well-being (13). It covers both the hedonic and eudaimonic aspects of mental health, including positive affects (feelings of optimism, cheerfulness, and relaxation), satisfying interpersonal relationships and positive functioning (energy, clear thinking, self-acceptance, personal development, competence, and autonomy). It has good content validity and high test–retest reliability. An overall higher score indicates more positive well-being. The WEMWBS has been shown to detect subtle change in populations with both good and poor mental health (56) and has already been widely used to study the COVID-19 outbreak (57, 58). For more details, see Supplementary methods.

#### 2.1.3. Predictors

We extracted the following variables:

##### 2.1.3.1. Background characteristics

- Socio-demographic factors: age; sex (male vs. female); being in a relationship (no vs. yes); educational attainment (up to 12 years of education (reference) vs. 12 to 14 years of education vs. 14 years of education to Bachelor level vs. Bachelor to Master’s level vs. Master’s to PhD level or above); working category (employee (reference) vs. self-employed vs. student vs. retiree); having undergone a lockdown period in the past (e.g., jail, long period of hospital admission: no vs. yes); having access to an outdoor space (e.g., balcony, private garden: no vs. yes); living area (living in an urban area (reference) vs. semi-urban area vs. rural area); having a pet (no vs. yes); social contacts, which we determined by frequency of casual face to face contacts, telephone calls, text messages and contacts from social networks (measured on Likert scales; Supplementary methods); living place [French districts (“*départements”*): 69 (Rhône) vs. 75 (Paris) vs. other (ref)].
- Health-related factors: having a chronic medical illness (no vs. yes); having a psychiatric history (no history (ref) vs. past history only vs. current issues). The latter was evaluated through the following question: “Have you ever been followed for a psychiatric and/or addiction problem (by a psychologist/psychiatrist/addiction specialist)?” Possible answers: yes currently, yes in the past, no never.

##### 2.1.3.2. COVID-related factors

- COVID-19 status and risk of contamination: COVID-19 negative and no contact with people (reference) vs. COVID-19 negative and in contact with people who are not contaminated vs. COVID-19 positive or COVID-19 negative and in contact with people who are contaminated or suspected to be contaminated;
- Lockdown policies and official information: agreement with lockdown measures; satisfaction with COVID-related information; satisfaction with the clarity of information provided by the government;
- Worries about negative consequences of lockdown: worries about having access to PPE; worries about having access to essential products; worries about financial consequences; worries about being in a precarious situation.

Lockdown policies and official information, as well as worries about negative consequences of lockdown, were measured on Likert scales (Supplementary methods).

##### 2.1.3.3. Coping strategies

- Support from … (no vs. yes): people living under the same roof; family (other than those living under the same roof); friends (other than those living under the same roof); colleagues; neighbors/acquaintances;
- Coping with … (no vs. yes): words from people around; beliefs in a favorable outcome; advances in knowledge and scientific progress; religious faith; resilience and past experiences; collective actions; beliefs in beneficial impact that lockdown can have on the planet; beliefs in positive impacts of lockdown on the individual; none of these.

Continuous predictors were variables with a numerical outcome (age) and variables measured on a Likert scale (e.g., frequency of contact). Other predictors were categorical.

Note that from the original sample (*N* = 19,205), we excluded participants who did not live in France, who were younger than 16 years-old or older than 75 years-old, and those who did not define themselves as male or female. Our final dataset included *N* = 18,957 observations.

The research board of the Vinatier Hospital (Bron, France) stated that no ethics committee approval was needed and that the project was conducted in accordance with survey ethics. Indeed, as the survey was conducted anonymously with no personal data the EU General Data Protection Regulation (GDPR) of May 25, 2018 did not apply.

### 2.2. Analysis

To predict well-being over the two-months lockdown period based on our large set of predictors, we ran a regression model, including main terms as well as their first order interaction with days since the survey was launched. Main effects identify overall effects of predictors (i.e., aggregated over the study period). Interaction effects identify whether the positive or negative effect of a given predictor significantly increases with time since the survey was launched.

We were facing two methodological constraints. First, we had a very large set of predictors, incurring the risk of multi-collinearity and unreliable coefficient estimates due to high variance. We addressed this challenge by running ridge regularization regression instead of standard (OLS) linear regression (59). Ridge regression, or L2 regularization, is a method of choice when analyzing data that contain a high number of predictors and/or that potentially suffer from multi-collinearity. Ridge regression adds a penalty to the coefficient estimates, shrinking less important estimates and making the variables less correlated, overall differentiating “important” from “less-important” predictors. Of note, ridge regression does not eliminate coefficients, as is the case in LASSO regression (L1 regularization).

Mathematically, ridge regression is adding a penalty parameter to the objective function of a standard linear model. Ridge regression aims to minimize (∥Xβ − y∥^2^ + λ∥β∥^2^), X being the matrix of independent variables, β being a vector of their parameter estimates, y being the vector of the dependent variable, and λ (lambda) being the penalization parameter. While coefficients estimated by the ridge estimator have lower variance compared to the OLS estimator, they may also suffer from a higher bias. The usual practice is to minimize the bias-variance trade-off by choosing an optimal penalty parameter using a cross-validation procedure (see below).

A second methodological constraint was the imbalance in the distribution of survey responses over the duration of lockdown (11,194 respondents in week 2; 5,008 in week 3; 629 respondents in week 4; 1,259 respondents in week 5; 394 respondents in week 6; 337 respondents in week 7; and 136 respondents in week 8). To address this caveat, we used a bootstrap down-sampling strategy. We ran the ridge algorithm 1,000 times after having down-sampled the data (with replacement) so that each lockdown week presented an equal number of observations, equal to the size of the minority class data (data from week 8). Random sampling with replacement was used to down-sample for the majority classes. Data from week 8 were also sampled with replacement but kept their original size. Each sample included N = 952 observations.

Using the R package *glmnet* (60), we trained each of the 1,000 samples using a k = 20 folds cross-validation procedure. Briefly, for each of the 20 folds, the procedure ran the ridge algorithm 100 times (each time with a different lambda value) on a training set including 95% of the data, and tested the predictive accuracy on an independent testing set including 5% of the data. The model with the best lambda value (i.e., where the R^2^ was maximized) was then selected and ran on the entire sample.

Overall, we obtained 1,000 best penalty parameters lambdas that each time maximized an R^2^, as well as 1,000 random cases of parameter estimates for each predictor. For each parameter, we therefore obtained a distribution of these randomly generated coefficients, from which we extracted our average estimate B (defined as the median of the distribution) and 95% confidence interval (2.5 and 97.5 percentiles). Parameter estimates whose 95% confidence intervals did not include zero were deemed as significant.

Pre-processing involved the exclusion of “near zero-variance” predictors, and of highly correlated predictors. Specifically, if two variables had a correlation greater than 0.9, we removed the variable with the largest mean absolute correlation. Note that no methods such as weighting or propensity scores were used to adjust for the non-representativeness of the sample to the French population.

Analyses were conducted with R 4.0.1, and packages *glmnet* (60) and *caret* (61).

## 3. Results

### 3.1. Data description

A total of 18,957 participants had a complete questionnaire (Table 1). 76.7% were women. 26% were aged 16–29 years, 47.5% were aged 30–49 years, 26.4% were aged 50 years and older. The majority of respondents were employed (66.3%), in a relationship (63.9%), had at least 14 years of education (80.4%), and were living in an urban area (54.1%). 74.8% of participants had no past or current psychiatric history.

**Table 1:** Socio-demographic and health-related characteristics of the participants.

Variables	Week 2 (*N* = 11,194)	Week 3 (*N* = 5,008)	Week 4 (*N* = 629)	Week 5 (*N* = 1,259)	Week 6 (*N* = 394)	Week 7 (*N* = 337)	Week 8 (*N* = 136)	Total (*N* = 18,957)
WEMWBS total score: mean (SD)	49.4 (8.1)	49.7 (7.9)	48.6 (8.8)	47.8 (8.7)	47.7 (9.7)	47.4 (8.9)	45.7 (10.3)	49.2 (8.2)
Sex								
Male	2,518 (22.5%)	1,238 (24.7%)	160 (25.4%)	297 (23.6%)	96 (24.4%)	68 (20.2%)	36 (26.5%)	4,413 (23.3%)
Female	8,676 (77.5%)	3,770 (75.3%)	469 (74.6%)	962 (76.4%)	298 (75.6%)	269 (79.8%)	100 (73.5%)	14,544 (76.7%)
Age								
16–29 y/o	3,350 (29.9%)	944 (18.8%)	123 (19.6%)	292 (23.2%)	117 (29.7%)	80 (23.7%)	30 (22.1%)	4,936 (26.0%)
30–49 y/o	5,274 (47.1%)	2,487 (49.7%)	294 (46.7%)	562 (44.6%)	171 (43.4%)	161 (47.8%)	64 (47.1%)	9,013 (47.5%)
50–74 y/o	2,570 (23.0%)	1,577 (31.5%)	212 (33.7%)	405 (32.2%)	106 (26.9%)	96 (28.5%)	42 (30.9%)	5,008 (26.4%)
In a relationship								
No	3,940 (35.2%)	1810 (36.1%)	238 (37.8%)	523 (41.5%)	140 (35.5%)	133 (39.5%)	62 (45.6%)	6,846 (36.1%)
Yes	7,254 (64.8%)	3,198 (63.9%)	391 (62.2%)	736 (58.5%)	254 (64.5%)	204 (60.5%)	74 (54.4%)	12,111 (63.9%)
Education								
Up to 12 years of education	1988 (17.8%)	736 (14.7%)	111 (17.6%)	171 (13.6%)	60 (15.2%)	51 (15.1%)	27 (19.9%)	3,144 (16.6%)
12 to 14 years of education	1,498 (13.4%)	667 (13.3%)	93 (14.8%)	167 (13.3%)	59 (15.0%)	44 (13.1%)	19 (14.0%)	2,547 (13.4%)
14 years to Bachelor level	2,435 (21.8%)	1,020 (20.4%)	132 (21.0%)	249 (19.8%)	76 (19.3%)	53 (15.7%)	27 (19.9%)	3,992 (21.1%)
Bachelor to Masters	4,157 (37.1%)	1972 (39.4%)	241 (38.3%)	555 (44.1%)	153 (38.8%)	163 (48.4%)	53 (39.0%)	7,294 (38.5%)
Masters to PhD or above	1,116 (10.0%)	613 (12.2%)	52 (8.3%)	117 (9.3%)	46 (11.7%)	26 (7.7%)	10 (7.4%)	1980 (10.4%)
Access to outdoor space								
No	2001 (17.9%)	787 (15.7%)	89 (14.1%)	226 (18.0%)	65 (16.5%)	52 (15.4%)	19 (14.0%)	3,239 (17.1%)
Yes	9,193 (82.1%)	4,221 (84.3%)	540 (85.9%)	1,033 (82.0%)	329 (83.5%)	285 (84.6%)	117 (86.0%)	15,718 (82.9%)
Work situation								
Employee	7,388 (66.0%)	3,415 (68.2%)	384 (61.0%)	840 (66.7%)	260 (66.0%)	212 (62.9%)	76 (55.9%)	12,575 (66.3%)
Self-employed	1,165 (10.4%)	536 (10.7%)	65 (10.3%)	117 (9.3%)	25 (6.3%)	29 (8.6%)	16 (11.8%)	1953 (10.3%)
Student	1,390 (12.4%)	441 (8.8%)	70 (11.1%)	156 (12.4%)	65 (16.5%)	40 (11.9%)	21 (15.4%)	2,183 (11.5%)
Retired	763 (6.8%)	446 (8.9%)	74 (11.8%)	94 (7.5%)	21 (5.3%)	26 (7.7%)	11 (8.1%)	1,435 (7.6%)
Other	488 (4.3%)	170 (3.4%)	36 (5.7%)	52 (4.1%)	23 (5.8%)	30 (8.9%)	12 (8.8%)	811 (4.3%)
Ever been locked								
No	8,922 (79.7%)	4,044 (80.8%)	489 (77.7%)	966 (76.7%)	311 (78.9%)	269 (79.8%)	109 (80.1%)	15,110 (79.7%)
Yes	2,272 (20.3%)	964 (19.2%)	140 (22.3%)	293 (23.3%)	83 (21.1%)	68 (20.2%)	27 (19.9%)	3,847 (20.3%)
Chronic medical pb								
No	9,446 (84.4%)	4,199 (83.8%)	523 (83.1%)	1,033 (82.0%)	318 (80.7%)	275 (81.6%)	105 (77.2%)	15,899 (83.9%)
Yes	1748 (15.6%)	809 (16.2%)	106 (16.9%)	226 (18.0%)	76 (19.3%)	62 (18.4%)	31 (22.8%)	3,058 (16.1%)
Psychiatric history								
No	8,386 (74.9%)	3,850 (76.9%)	448 (71.2%)	898 (71.3%)	289 (73.4%)	216 (64.1%)	86 (63.2%)	14,173 (74.8%)
Past psychiatric history	1,602 (14.3%)	658 (13.1%)	101 (16.1%)	199 (15.8%)	49 (12.4%)	48 (14.2%)	21 (15.4%)	2,678 (14.1%)
Current psychiatric issues	1,206 (10.8%)	500 (10.0%)	80 (12.7%)	162 (12.9%)	56 (14.2%)	73 (21.7%)	29 (21.3%)	2,106 (11.1%)
Area of living								
Urban area	6,192 (55.3%)	2,616 (52.2%)	317 (50.4%)	681 (54.1%)	203 (51.5%)	173 (51.3%)	71 (52.2%)	10,253 (54.1%)
Semi-rural area	2,373 (21.2%)	1,101 (22.0%)	158 (25.1%)	286 (22.7%)	98 (24.9%)	76 (22.6%)	34 (25.0%)	4,126 (21.8%)
Rural area	2,629 (23.5%)	1,291 (25.8%)	154 (24.5%)	292 (23.2%)	93 (23.6%)	88 (26.1%)	31 (22.8%)	4,578 (24.1%)
Having a pet								
No	5,845 (52.2%)	2,793 (55.8%)	351 (55.8%)	711 (56.5%)	215 (54.6%)	192 (57.0%)	84 (61.8%)	10,191 (53.8%)
Yes	5,349 (47.8%)	2,215 (44.2%)	278 (44.2%)	548 (43.5%)	179 (45.4%)	145 (43.0%)	52 (38.2%)	8,766 (46.2%)

The overall mean WEMWBS total score was 49.2 (sd 8.2). There were variations depending on the week of lockdown, starting at 49.4 (sd 8.1) in week 2, then 49.7 (sd 7.9) in week 3, followed by a gradual decrease to 45.7 (sd 10.3) in week 8 (Table 1). Please refer to Table 1 for a description of other socio-demographic and health-related characteristics of the participants throughout the study period. Please refer to Supplementary Table 1 for a description of the remaining predictors.

### 3.2. Adjusted analysis

We then randomly down-sampled the data 1,000 times to have a balanced representation of each of the 7 lockdown weeks. We subsequently ran a ridge regression of the above-mentioned predictors on the WEMWBS total score in each of the 1,000 sampled data. Over the 1,000 samples, the range of the penalty parameter lambda was [0.32, 6.05], and the range of R^2^ was [0.29, 0.46]. Mean R^2^ was of 0.37. Below, we report significant main effects (overall effects over the study period) as well as significant temporal effects (positive and negative effects that increase with time spent in lockdown) for background characteristics, COVID-related factors and support/coping strategies.

#### 3.2.1. Main effects

**Figure 1:**
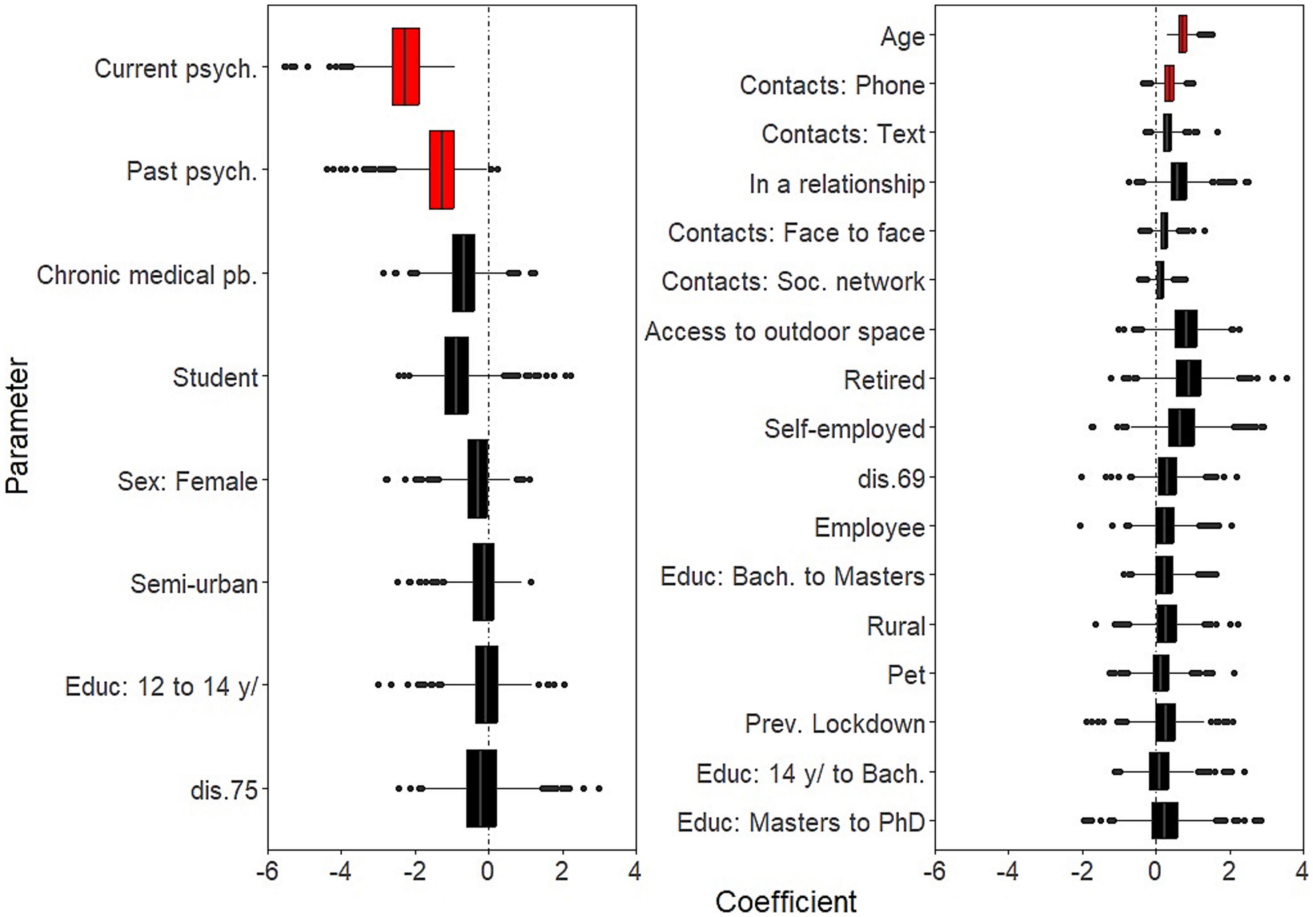
Effects of background characteristics on mental well-being. Boxplots of the bootstrap estimates are displayed for all parameters. Boxplots visualize five summary statistics: the median, two hinges and two whiskers. The lower and upper hinges correspond to the first and third quartiles (the 25th and 75th percentiles). The upper whisker extends from the hinge to the largest value no further than 1.5 * IQR from the hinge (where IQR is the inter-quartile range, or distance between the first and third quartiles). The lower whisker extends from the hinge to the smallest value at most 1.5 * IQR of the hinge. Data beyond the end of the whiskers are plotted individually. Each estimate represents the effect of each predictor over the entire study period (7 weeks of lockdown). Average estimates (median of the bootstrap distribution) that are negative are on the left and those that are positive are on the right. Significant estimates (whose 95% confidence interval do not include zero) are in red. Legend. Bach., Bachelor level; dis., district; psych., psychiatric issues; pb., problem; Educ, Education; Soc., Social; Prev., Previous.

**Figure 2:**
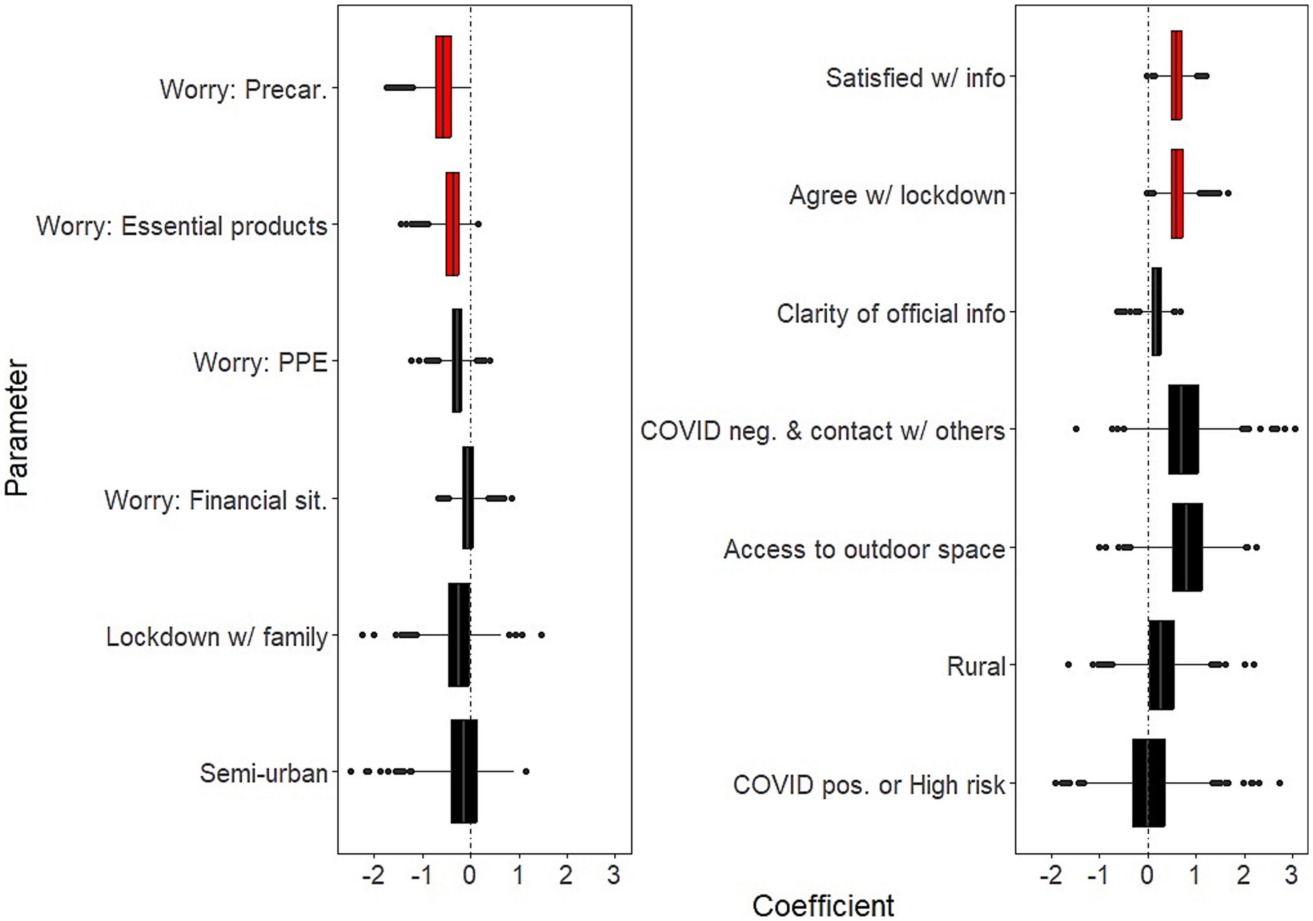
Effects of COVID-related factors on mental well-being. Boxplots of the bootstrap estimates are displayed for all parameters. Boxplots visualize five summary statistics: the median, two hinges and two whiskers. The lower and upper hinges correspond to the first and third quartiles (the 25th and 75th percentiles). The upper whisker extends from the hinge to the largest value no further than 1.5 * IQR from the hinge (where IQR is the inter-quartile range, or distance between the first and third quartiles). The lower whisker extends from the hinge to the smallest value at most 1.5 * IQR of the hinge. Data beyond the end of the whiskers are plotted individually. Each estimate represents the effect of each predictor over the entire study period (7 weeks of lockdown). Average estimates (median of the bootstrap distribution) that are negative are on the left and those that are positive are on the right. Significant estimates (whose 95% confidence interval do not include zero) are in red. Legend. PPE, Personal Protective Equipment; Precar., Precariousness; sit., situation; info, COVID-related information; neg., negative; pos., positive.

**Figure 3:**
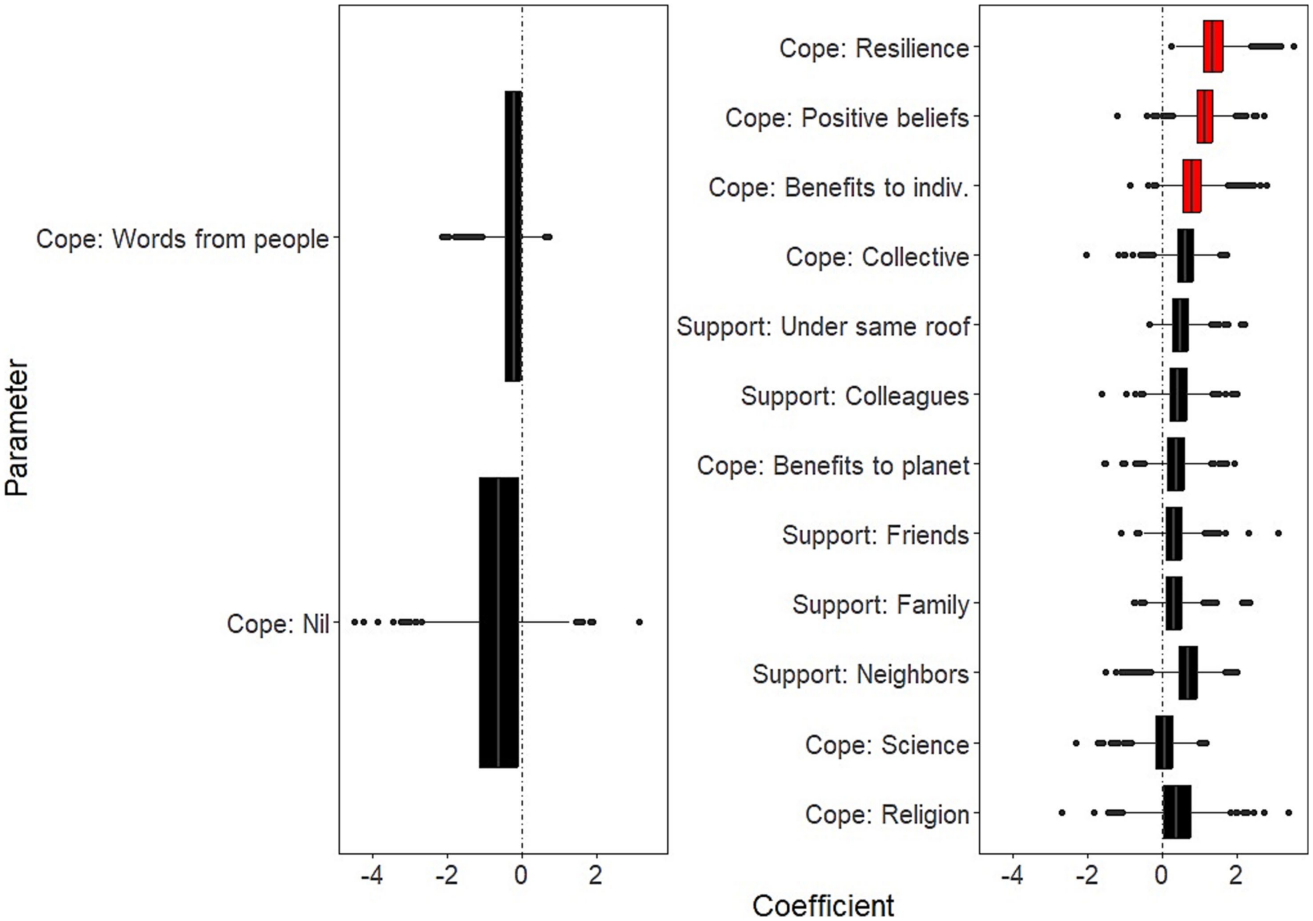
Effects of coping strategies on mental well-being. Boxplots of the bootstrap estimates are displayed for all parameters. Boxplots visualize five summary statistics: the median, two hinges and two whiskers. The lower and upper hinges correspond to the first and third quartiles (the 25th and 75th percentiles). The upper whisker extends from the hinge to the largest value no further than 1.5 * IQR from the hinge (where IQR is the inter-quartile range, or distance between the first and third quartiles). The lower whisker extends from the hinge to the smallest value at most 1.5 * IQR of the hinge. Data beyond the end of the whiskers are plotted individually. Each estimate represents the effect of each predictor over the entire study period (7 weeks of lockdown). Average estimates (median of the bootstrap distribution) that are negative are on the left and those that are positive are on the right. Significant estimates (whose 95% confidence interval do not include zero) are in red. Legend. indiv., the individual.

- Background socio-demographic and health-related characteristics that were significantly associated with decreased well-being over the study period were: having current psychiatric issues (B = −2.26; 95% CI: −3.82, −1.39); having past psychiatric problems (B = −1.27; 95% CI: −2.75, −0.38) (Figure 1, left panel and Supplementary Table 2). Background characteristics that were significantly associated with increased well-being were age (B = 0.71 for each additional 10 years of age; 95% CI: 0.47, 1.14); and social contacts (as measured by phone contacts: B = 0.36; 95% CI: 0.04, 0.69) (Figure 1, right panel and Supplementary Table 2).
- COVID-related factors that were significantly associated with decreased well-being over the study period were: worries about being in a precarious situation (B = −0.57; 95% CI: −1.22, −0.16); worries about having access to essential products (B = −0.37; 95% CI: −0.89, −0.04; Figure 2, left panel and Supplementary Table 3). COVID-related factors that were significantly associated with increased well-being were: satisfaction with COVID-related information (B = 0.60; 95%CI: 0.31, 0.97); agreement with lockdown measures (B = 0.60; 95%CI: 0.25, 1.04; Figure 2, right panel and Supplementary Table 3).
- Coping strategies associated with increased well-being over the study period were: resilience (B = 1.35; 95%CI: 0.75, 2.34); beliefs in a favorable outcome (B = 1.14; 95%CI: 0.48, 1.83); beliefs that lockdown has positive impacts on the individual (B = 0.78; 95%CI: 0.16, 1.65; Figure 3, right panel and Supplementary Table 4). There were no coping strategies that had a significantly negative effect on well-being (Figure 3, left panel and Supplementary Table 4).

#### 3.2.2. Temporal effects

**Figure 4:**
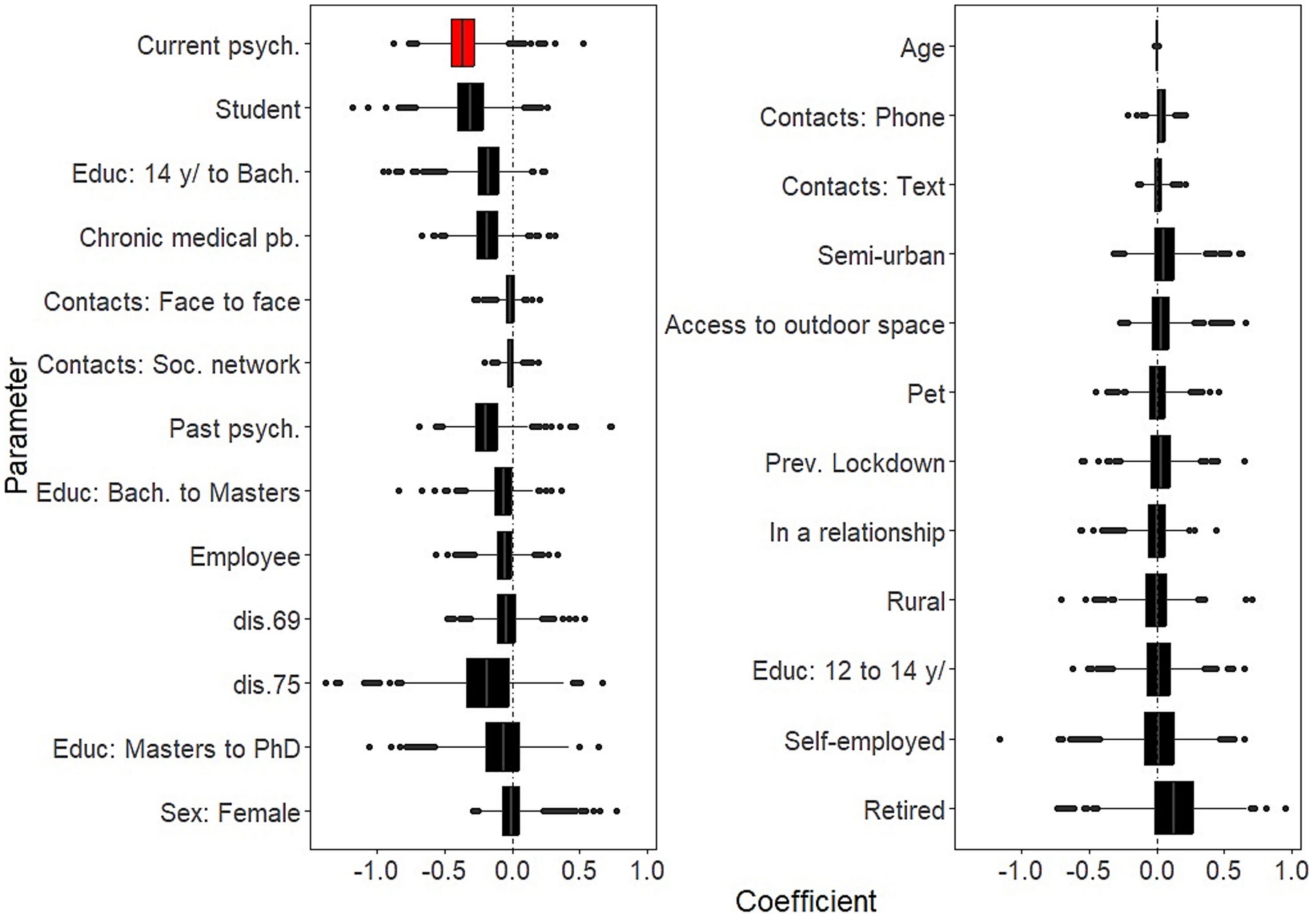
Temporal effects of background characteristics on mental well-being. Boxplots of the bootstrap estimates are displayed for all parameters. Boxplots visualize five summary statistics: the median, two hinges and two whiskers. The lower and upper hinges correspond to the first and third quartiles (the 25th and 75th percentiles). The upper whisker extends from the hinge to the largest value no further than 1.5 * IQR from the hinge (where IQR is the inter-quartile range, or distance between the first and third quartiles). The lower whisker extends from the hinge to the smallest value at most 1.5 * IQR of the hinge. Data beyond the end of the whiskers are plotted individually. Each estimate represents the effect of each predictor for 10 days of lockdown. Average estimates (median of the bootstrap distribution) that are negative are on the left and those that are positive are on the right. Significant estimates (whose 95% confidence interval do not include zero) are in red. Legend. Bach., Bachelor level; dis., district; psych., psychiatric issues; pb., problem; Educ, Education; Soc., Social; Prev., Previous.

**Figure 5:**
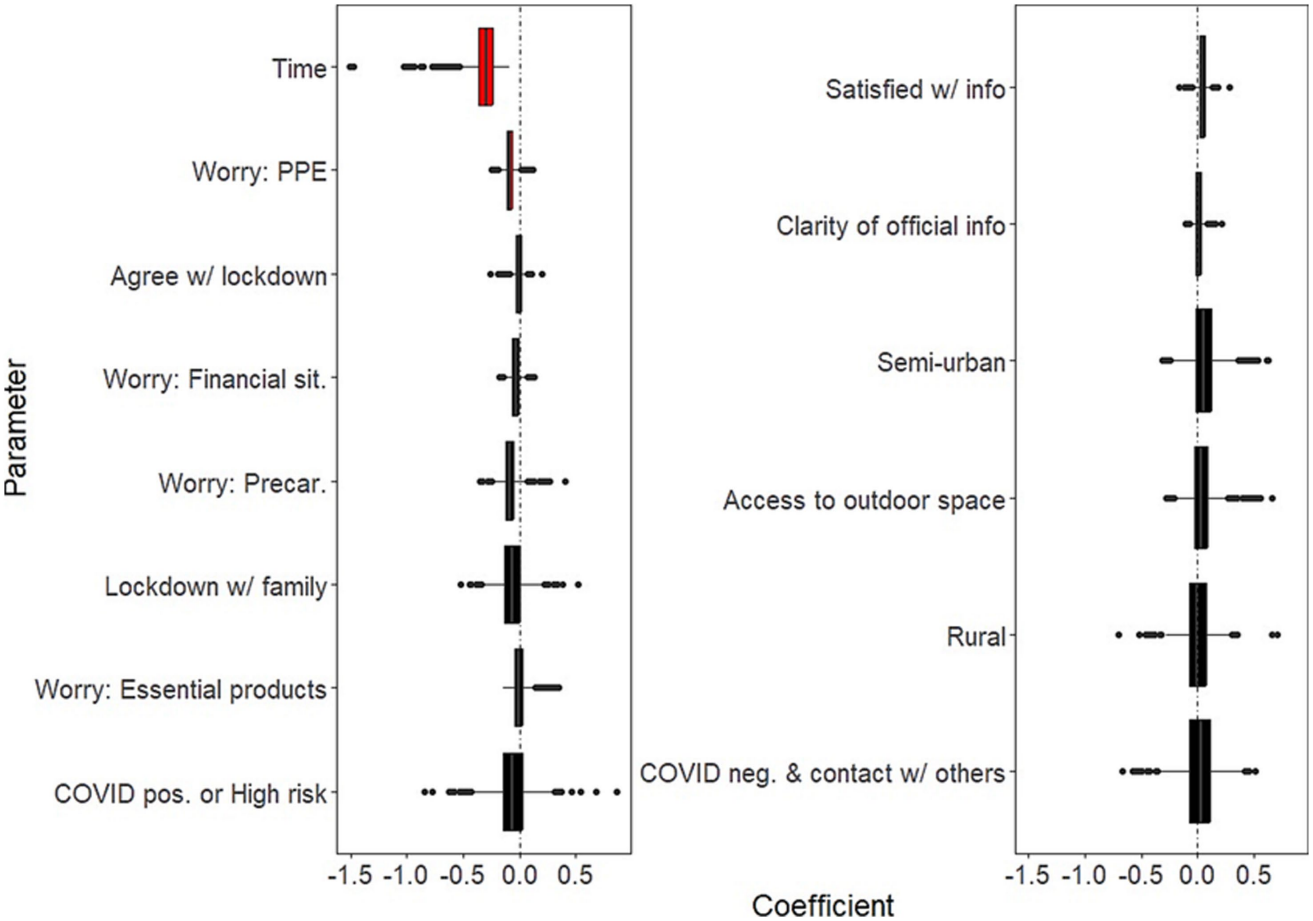
Temporal effects of COVID-related factors on mental well-being. Boxplots of the bootstrap estimates are displayed for all parameters. Boxplots visualize five summary statistics: the median, two hinges and two whiskers. The lower and upper hinges correspond to the first and third quartiles (the 25th and 75th percentiles). The upper whisker extends from the hinge to the largest value no further than 1.5 * IQR from the hinge (where IQR is the inter-quartile range, or distance between the first and third quartiles). The lower whisker extends from the hinge to the smallest value at most 1.5 * IQR of the hinge. Data beyond the end of the whiskers are plotted individually. Each estimate represents the effect of each predictor for 10 days of lockdown. Average estimates (median of the bootstrap distribution) that are negative are on the left and those that are positive are on the right. Significant estimates (whose 95% confidence interval do not include zero) are in red. Legend. PPE, Personal Protective Equipment; Precar., Precariousness; sit., situation; info, COVID-related information; neg., negative; pos., positive.

**Figure 6:**
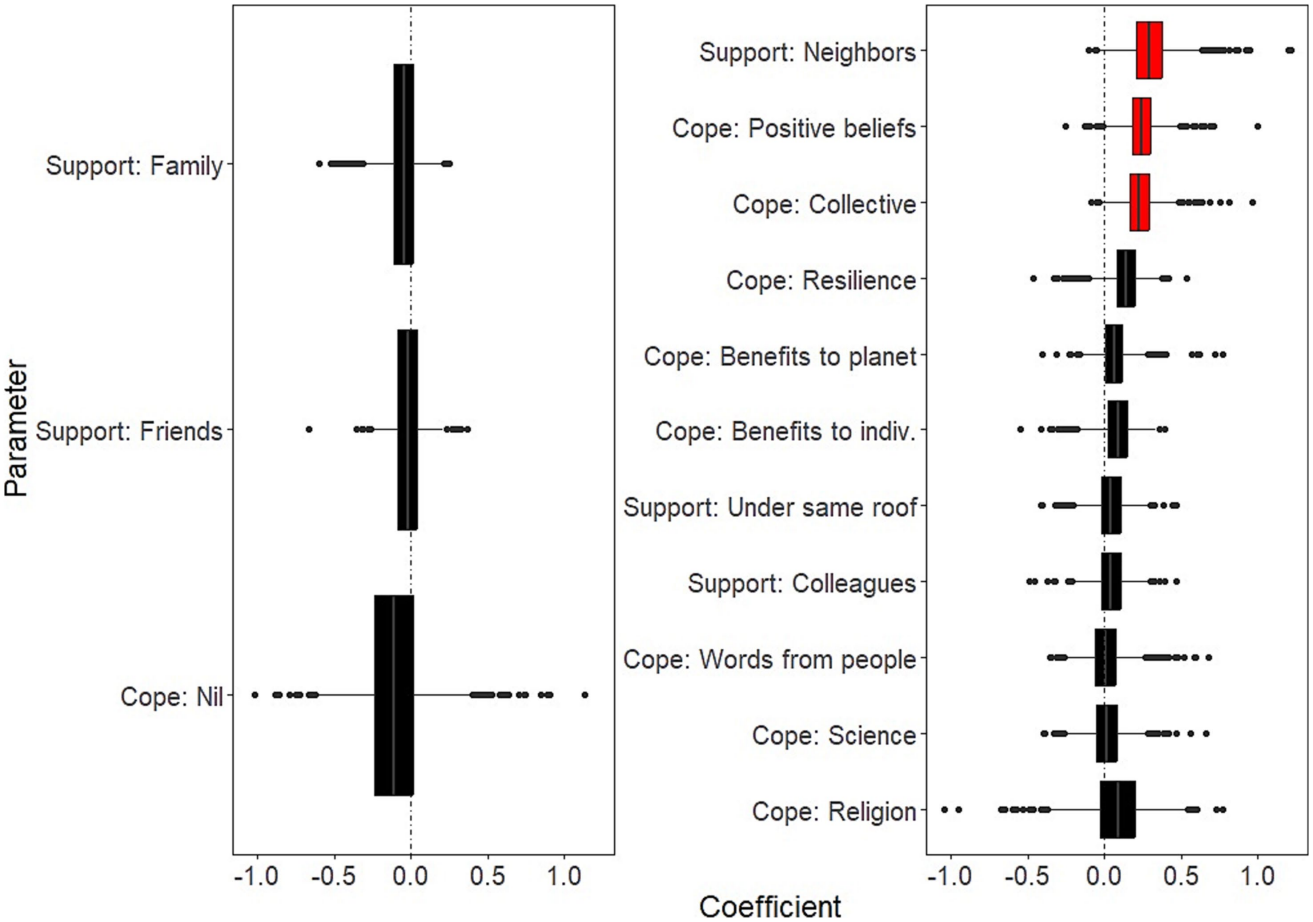
Temporal effects of coping strategies on mental well-being. Boxplots of the bootstrap estimates are displayed for all parameters. Boxplots visualize five summary statistics: the median, two hinges and two whiskers. The lower and upper hinges correspond to the first and third quartiles (the 25th and 75th percentiles). The upper whisker extends from the hinge to the largest value no further than 1.5 * IQR from the hinge (where IQR is the inter-quartile range, or distance between the first and third quartiles). The lower whisker extends from the hinge to the smallest value at most 1.5 * IQR of the hinge. Data beyond the end of the whiskers are plotted individually. Each estimate represents the effect of each predictor for 10 days of lockdown. Average estimates (median of the bootstrap distribution) that are negative are on the left and those that are positive are on the right. Significant estimates (whose 95% confidence interval do not include zero) are in red. Legend. indiv., the individual.

Days in lockdown was a significant predictor of negative well-being (for 10 additional days since the survey was launched: B = −0.30, 95%CI: −0.62, −0.15). In addition:

- Background socio-demographic and health-related characteristics that showed significantly negative effects on well-being as time in lockdown progressed were: having current psychiatric issues (B = −0.37; 95%CI: −0.63, −0.04; Figure 4, left panel and Supplementary Table 5). There was no background characteristics that showed significantly positive effects on well-being as time in lockdown progressed (Figure 4, right panel and Supplementary Table 5).
- COVID-related factors that showed significantly negative effects on well-being as time in lockdown progressed were: worries about having access to PPE (B = −0.09; 95%CI: −0.18, −0.01) (Figure 5, left panel and Supplementary Table 6). There were no COVID-related factors that showed significantly positive effects on well-being as time in lockdown progressed (Figure 5, right panel and Supplementary Table 6).
- Coping strategies that showed significantly negative effects on well-being as time in lockdown progressed were: beliefs in a favorable outcome (B = 0.29; 95%CI: 0.04, 0.62); support by neighbors (B = 0.24; 95%CI: 0.04, 0.44); and collective actions (B = 0.23; 95%CI: 0.04, 0.46) (Figure 6, right panel and Supplementary Table 7). There were no significantly coping strategies that showed negative effects on well-being as time in lockdown progressed (Figure 6, left panel and Supplementary Table 7).

## 4. Discussion

Although social distancing did help to contain the spread of COVID-19, this strategy had a major impact on the population’s mental health. This study aimed to investigate the effects of a large number of background characteristics, COVID-related factors, and coping strategies on mental well-being in a sample of the French population during seven of the 8 weeks of the first lockdown due to the COVID-19 pandemic.

We found that factors that were significantly associated with decreased well-being over the study period were: having current and past psychiatric issues, having worries about being in a precarious situation and about having access to essential products. Factors that were significantly associated with increased well-being were: age, social contacts pre-pandemic period, satisfaction with COVID-related information, agreement with lockdown measures, resilience, beliefs in a favorable outcome and in positive impacts of lockdown on the individual.

Moreover, time in lockdown was associated with a decrease in well-being. Having current psychiatric issues and having worries about having access to personal protective equipment showed significantly negative effects on well-being as time in lockdown progressed. In contrast, coping by having positive beliefs about the future of the pandemic, being supported by neighbors, and being involved in collective actions showed significantly positive effects on well-being as time in lockdown progressed.

Below, we discuss significant effects from these three categories of predictors (background characteristics, COVID-related factors, and coping strategies). We then discuss temporal effects, that is, positive and negative effects on well-being as time in lockdown progressed.

### 4.1. Background characteristics

We found that having less regular contacts with people before lockdown were associated with lower well-being whilst in lockdown. Loneliness has long been shown to be related to poor mental health outcomes (62), an increased risk of depression (63), but also increased physical health problems and mortality (64). Yet, while social distancing may have affected loneliness (29, 65), others have suggested that the effects of loneliness on mental well-being were for the most part precedent to lockdown (66, 67). This may explain why having less regular contact with people *whilst in lockdown* was not retrieved as a significant factor in our study.

Those with a current or past psychiatric illness may also be particularly sensitive to social distancing, as shown in previous reports (47). This may be due to excessive fear of the virus or to increased difficulties to deploy diverting strategies to cope with life. This population may also be particularly exposed to the negative consequences of social distancing with their mental health teams.

Being of a young age also showed a negative effect on well-being. Time in lockdown may have negatively impacted young persons more than older adults as the former may have felt more constrained and less autonomous due to social and movement restrictions during the pandemic (68). Connecting with others is critical to psychological development (69–72), and any restrictions in social connectedness (e.g., due to social distancing) might alter well-being (73). Young persons may also be particularly affected by the pandemic, due to higher risks of economic difficulties (27). In addition, it has been shown that while older individuals were more concerned by COVID-19 at the very beginning of the pandemic, they were less worried about the virus during lockdown and changed their behavior to a lesser extent (74).

As mentioned in the Introduction, others have shown that female gender was associated with decreased well-being (15–17), yet this relationship did not turn out to be significant in our study. Differences in sample selection may explain these discrepancies, given that individuals from our sample have voluntarily chosen to be involved in the study. Another potential explanation is that we investigated a large number of predictors that may be linked to gender (e.g., having psychiatric issues, worrying about being in a precarious situation or about having access to essential products). These in turn may have masked the effect of gender on well-being.

### 4.2. COVID-related factors

COVID-related policies resulted in citizens having to face the deterioration of economic conditions. Similarly to others, we showed that worries about the breakdown of supply chains of essential products or PPE, and worries about being in a precarious situation (e.g., due to work restrictions), were significantly associated with lower levels of well-being (35, 75). As obvious as they may seem, these findings underline the absolute necessity for policymakers to provide all efforts to ensure the supply of basic goods that are critical to one’s survival and protection.

Disagreement with lockdown policies and dissatisfaction with information provided about the pandemic were also associated with lower well-being. At their most extreme, citizens with lower levels of well-being may demonstrate resentment and mistrust for governments and policy makers (37, 75), that themselves are correlated with COVID-19 and generic conspiracy and pseudo-scientific beliefs (37, 76).

Interestingly, effects of COVID-related stressors may be moderated by personality traits such as extroversion or emotional reactivity, which themselves have been found to predict well-being during the COVID-19 pandemic (68, 75, 77). For instance, it was demonstrated that lifestyle disruptions due to COVID-lockdown was less detrimental to introverts than extroverts (68, 77), perhaps because the former were more adapted to lockdown life circumstances than the latter. Further investigation is needed to disentangle the effect of personality traits vs. contextual factors (e.g., economic risk, supply chains and agreement with policies) on overall levels of well-being.

### 4.3. Coping strategies

In line with others, we found that coping strategies that specifically demonstrated an effect on well-being during lockdown involved collective actions (78) and having positive beliefs about the future (34). Research in Wuhan, China has shown that neighborhoods’ social infrastructure (e.g., services provided by urban residents’ committees and volunteer groups) provided social cohesion (78). In turn, such an increased sense of community and connectedness may mitigate the decreased level of well-being observed during lockdown (78, 79) in promoting eudaimonic living (80).

Likewise, we showed that optimistic beliefs about the future of the pandemic improved well-being and quality of life. We also found virtuous effects of beliefs in positive impacts of lockdown on the individual: spending less on fuel, working from home, or staying home with their loved ones. In line with our findings, some have shown that hope, optimism and acceptance, along with other psychological resources such as gratitude of being, gratitude towards the world, and personal wisdom, increased the level of well-being during COVID (48). Of note, others have pointed that at their extremes, such psychological traits may also be somehow unrealistic and at the cost of underestimation of health risk (75, 81, 82).

Finally, those having skills to adapt to difficult situations (a.k.a. resilience) showed an obvious advantage in terms of well-being during lockdown. Resilience however is closely linked to the concept of agency, “the capacity to make choices and the power to act on those choices” (83), which by definition is seriously compromised during lockdown periods. Key domains in relation to successful coping whilst in lockdown were suggested to depend on actively and intentionally being involved in physical health, spiritual health, and social connection (29). In contrast, avoidant coping strategies, such as self-distraction, venting, denial, and emotional disengagement, were shown to be associated with negative health outcomes, such as increased loneliness (84).

### 4.4. Temporal effects

Our analytical framework allowed to disentangle overall main effects from those that linearly changed with time spent in lockdown. We reasoned that the latter effects may be associated with the so-called “pandemic fatigue.” According to the WHO, “pandemic fatigue is an expected and natural response to […] the implementation of invasive measures” to curb the spread of the virus (53). Pandemic fatigue is “defined as de-motivation to follow recommended protective behaviors, emerging gradually over time and affected by a number of emotions, experiences and perceptions” (53). As suggested, lockdown may be both psychologically demanding and “cost accumulating,” with gradual increase in psychological fatigue associated with physical distancing (85). In other words, one of the major contributors of pandemic fatigue may be the gradual deterioration of well-being as a result of lockdown policies (86). In our study, overall well-being deteriorated as time in lockdown progressed, and even more so after the seventh week of lockdown. Those factors that showed negative effects on well-being as time in lockdown progressed may therefore predispose or precipitate to pandemic fatigue: having current psychiatric issues and worrying about having access to PPE. By contrast, factors that showed positive effects on well-being as time in lockdown progressed may be protective of pandemic fatigue: having support from neighbors, coping by having positive beliefs about the future, coping by being involved in collective actions. We wish to encourage policymakers to better consider these factors to prevent any de-motivation to follow policies, in case more social distancing becomes necessary in the future.

### 4.5. Limitations and strengths

Our study has several limitations. First, individuals from our sample have voluntarily chosen to be involved in the study, therefore we cannot generalize our results to the French population. For instance, our sample is likely to under-estimate the mental health effects of lockdown, as those who are digitally excluded may be under-represented.

Second, participation dropped significantly after the second week of lockdown, and even more so after the third week. While we used a down-sample strategy to address this caveat, in an ideal situation we would not have observed such a dramatic drop of the number of observations.

Third, a longitudinal design investigating well-being pre- and post-lockdown would have been more appropriate than a repeated cross-sectional design investigating well-being during lockdown. While we cannot ascertain causality, our study still provides an interesting description of predictive factors of well-being during lockdown, and may be used as background knowledge for further research.

Fourth, our results may not be generalizable to other periods of COVID lockdowns. Indeed, passed the first wave, individuals may have gotten used to the pandemic and may have reacted differently to government restrictions. Therefore, our results need to be interpreted with respect to the *initial* lockdown period.

Fifth, our model only included main effects and first order interactions with time in lockdown. We were therefore unable to discuss potential interactions between predictors and non-linear temporal effects. For instance, the first announcement by the French government on 16 March set the duration of lockdown at 2 weeks. This was then extended on 27 March for another 2 weeks, before a final 4-week extension was announced on 13 April. This series of official announcements may have had an impact on mental well-being, which may have translated into non-linear temporal effects.

Sixth, psychological well-being was the only mental health-related construct assessed in this study. Therefore, our results should not be interpreted with respect to other dimensions of well-being (e.g., physical or social well-being). Likewise, other psychopathological domains, such as the emergence of affective symptoms, were shown to be significantly impacted by the COVID-19 pandemic (5). It would have been interesting to perform our analysis on other psychopathological constructs to investigate whether background characteristics, COVID-related factors, and coping strategies, were differentially associated with well-being vs. other measures of mental health.

Our study also has a number of strengths. First, the number of observations was high. Second, we investigated a large number of predictors of various nature (background characteristics, COVID-related factors, coping strategies). Third, we reduced the risk of multi-collinearity by running ridge regularization regression instead of standard (OLS) linear regression. Fourth, we palliated for the imbalance in the distribution of survey responses over the duration of lockdown using a bootstrap down-sampling strategy.

### 4.6. Conclusion

Despite those limitations, our data suggest that a substantial number of factors predicted deterioration of mental well-being over the first lockdown period due to the COVID-19 pandemic in France. Vulnerable populations were identified as young persons, people with less social contacts, and those having psychiatric issues. Unfortunately, there are risks of other pandemics like COVID-19 in the near future (87), with potential disastrous consequences on mental health. Our findings suggest that, in case of future lockdowns, financial and economic measures should protect those who are the most at risk of financial precariousness and at risk of not accessing basic goods. In addition, policy-makers should think of promoting other interventions, such as collective actions and local support (e.g., from neighbors). Not only would such interventions increase mental well-being during lockdown periods, and have positive spillover effects on related mental and physical health in the long-term. These interventions may also decrease the likelihood of pandemic fatigue and in turn, increase compliance to lockdown measures.

## Data availability statement

The raw data supporting the conclusions of this article will be made available by the authors, without undue reservation.

## Ethics statement

The requirement of ethical approval was waived by the research board of the Vinatier Hospital (Bron, France) for the studies involving humans. The studies were conducted in accordance with the local legislation and institutional requirements. The participants provided their written informed consent to participate in this study.

## Author contributions

FH, EZ, and NF conceived and designed the study. AT ran the first set of analyses and wrote the first draft. GB performed a second set of analyses and wrote the second draft in response to the major comments made by the reviewers. All authors provided an interpretation of the data and critical revisions to the manuscript. All authors read and approved the submitted manuscript. All authors had full access to the data (including statistical reports and tables) in the study and take responsibility for the integrity of the data and the accuracy of the data analysis.

## Conflict of interest

The authors declare that the research was conducted in the absence of any commercial or financial relationships that could be construed as a potential conflict of interest.

## Publisher’s note

All claims expressed in this article are solely those of the authors and do not necessarily represent those of their affiliated organizations, or those of the publisher, the editors and the reviewers. Any product that may be evaluated in this article, or claim that may be made by its manufacturer, is not guaranteed or endorsed by the publisher.
