# Supplementary material for: Predictors of mental well-being over the first lockdown period due to the COVID-19 pandemic in France. A repeated cross-sectional study
Source: Front Public Health. 2023 Aug 28;11:1234023. doi: 10.3389/fpubh.2023.1234023 (PMC10493269; doi:10.3389/fpubh.2023.1234023)
Supplement: Supplementary file 1 [file Data_Sheet_1.DOCX]

Supplementary Materials

Predictors of mental well-being over the first lockdown period due to the COVID-19 pandemic in France. A repeated cross-sectional study.

Guillaume Barbalat^†^, Audrey Tanguy Melac^†^, Elodie Zante, Frédéric Haesebaert, Nicolas Franck

^†^ These authors contributed equally to this work and share first authorship.

**Supplementary Methods.** Further description of the outcome and predictor variables. *(p.2)*

**Supplementary Table 1.** Description of background characteristics, COVID-related factors and coping strategies. *(p.3)*

**Supplementary Table 2.** Effects of background characteristics on mental well-being. *(p.8)*

**Supplementary Table 3.** Effects of COVID-related factors on mental well-being. *(p.9)*

**Supplementary Table 4.** Effects of coping strategies on mental well-being. *(p.10)*

**Supplementary Table 5.** Temporal effects of background characteristics on mental well-being. *(p.11)*

**Supplementary Table 6.** Temporal effects of COVID-related factors on mental well-being. *(p.12)*

**Supplementary Table 7.** Temporal effects of coping strategies on mental well-being. *(p.12)*

**Supplementary Methods**. **Further description of the outcome and predictor variables.**

*Outcome variable (Warwick-Edinburgh Mental Well-being Scale, WEMWBS)*

Each of the 14 items of the WEMWBS covers feeling and functioning aspects of mental well-being (e.g., “I’ve been feeling optimistic about the future”, “I’ve been feeling close to other people”). Participants are asked to rate the frequency of how they have experienced each statement over the past week from 1 (none of the time) to 5 (all of the time).

*Likert scales for the some of the predictors*

- Social contacts were determined by frequency of casual face to face contacts, telephone calls, text messages and contacts from social networks (measured on Likert scales: less than once a week < once a week < several times a week < every day);

- Lockdown policies and official information: agreement with lockdown measures (measured on a Likert scale: no < neutral < rather agree < completely agree); satisfaction with COVID-related information (Likert scale: very unsatisfied < rather not satisfied < neutral < rather satisfied < very satisfied); satisfaction with the clarity of information provided by the government (Likert scale: very unclear < not clear < neutral < rather clear < very clear);

- Worries about negative consequences of lockdown: worries about having access to PPE (measured on a Likert scale: no < rather not < rather yes < yes); worries about having access to essential products (not worried < worried but no problems accessing essential products < worried and problems accessing essential products); worries about financial consequences (Likert scale: certainly not < probably not < probably < very likely); worries about being in a precarious situation (Likert scale: certainly not < probably not < probably);

**Supplementary Table 1. Description of background characteristics*, COVID-related factors and coping strategies.**

| **Predictors** | Week 2 (N=11194) | Week 3 (N=5008) | Week 4 (N=629) | Week 5 (N=1259) | Week 6 (N=394) | Week 7 (N=337) | Week 8 (N=136) | Total (N=18957) |
| --- | --- | --- | --- | --- | --- | --- | --- | --- |
| **Contacts: Face to face** |  |  |  |  |  |  |  |  |
| Less than once a week | 1839 (16.4%) | 887 (17.7%) | 114 (18.1%) | 223 (17.7%) | 87 (22.1%) | 78 (23.1%) | 32 (23.5%) | 3260 (17.2%) |
| Once a week | 1786 (16.0%) | 805 (16.1%) | 99 (15.7%) | 199 (15.8%) | 70 (17.8%) | 65 (19.3%) | 23 (16.9%) | 3047 (16.1%) |
| Many times a week | 3637 (32.5%) | 1657 (33.1%) | 202 (32.1%) | 406 (32.2%) | 116 (29.4%) | 109 (32.3%) | 41 (30.1%) | 6168 (32.5%) |
| Everyday | 3932 (35.1%) | 1659 (33.1%) | 214 (34.0%) | 431 (34.2%) | 121 (30.7%) | 85 (25.2%) | 40 (29.4%) | 6482 (34.2%) |
| **Contacts: Phone** |  |  |  |  |  |  |  |  |
| Less than once a week | 1293 (11.6%) | 550 (11.0%) | 70 (11.1%) | 186 (14.8%) | 53 (13.5%) | 46 (13.6%) | 21 (15.4%) | 2219 (11.7%) |
| Once a week | 2213 (19.8%) | 1043 (20.8%) | 134 (21.3%) | 270 (21.4%) | 93 (23.6%) | 79 (23.4%) | 38 (27.9%) | 3870 (20.4%) |
| Many times a week | 4401 (39.3%) | 2123 (42.4%) | 261 (41.5%) | 526 (41.8%) | 154 (39.1%) | 141 (41.8%) | 49 (36.0%) | 7655 (40.4%) |
| Everyday | 3287 (29.4%) | 1292 (25.8%) | 164 (26.1%) | 277 (22.0%) | 94 (23.9%) | 71 (21.1%) | 28 (20.6%) | 5213 (27.5%) |
| **Contacts: Text** |  |  |  |  |  |  |  |  |
| Less than once a week | 654 (5.8%) | 335 (6.7%) | 47 (7.5%) | 103 (8.2%) | 40 (10.2%) | 36 (10.7%) | 20 (14.7%) | 1235 (6.5%) |
| Once a week | 840 (7.5%) | 390 (7.8%) | 46 (7.3%) | 131 (10.4%) | 43 (10.9%) | 37 (11.0%) | 16 (11.8%) | 1503 (7.9%) |
| Many times a week | 4003 (35.8%) | 2022 (40.4%) | 277 (44.0%) | 503 (40.0%) | 157 (39.8%) | 130 (38.6%) | 53 (39.0%) | 7145 (37.7%) |
| Everyday | 5697 (50.9%) | 2261 (45.1%) | 259 (41.2%) | 522 (41.5%) | 154 (39.1%) | 134 (39.8%) | 47 (34.6%) | 9074 (47.9%) |
| **Contacts: Soc. network** |  |  |  |  |  |  |  |  |
| Less than once a week | 2122 (19.0%) | 1299 (25.9%) | 167 (26.6%) | 369 (29.3%) | 121 (30.7%) | 115 (34.1%) | 47 (34.6%) | 4240 (22.4%) |
| Once a week | 762 (6.8%) | 353 (7.0%) | 36 (5.7%) | 116 (9.2%) | 33 (8.4%) | 26 (7.7%) | 9 (6.6%) | 1335 (7.0%) |
| Many times a week | 3058 (27.3%) | 1403 (28.0%) | 183 (29.1%) | 332 (26.4%) | 99 (25.1%) | 85 (25.2%) | 40 (29.4%) | 5200 (27.4%) |
| Everyday | 5252 (46.9%) | 1953 (39.0%) | 243 (38.6%) | 442 (35.1%) | 141 (35.8%) | 111 (32.9%) | 40 (29.4%) | 8182 (43.2%) |
| **Agree w/ lockdown** |  |  |  |  |  |  |  |  |
| No | 437 (3.9%) | 219 (4.4%) | 60 (9.5%) | 136 (10.8%) | 39 (9.9%) | 43 (12.8%) | 21 (15.4%) | 955 (5.0%) |
| Neutral | 544 (4.9%) | 286 (5.7%) | 59 (9.4%) | 166 (13.2%) | 39 (9.9%) | 26 (7.7%) | 17 (12.5%) | 1137 (6.0%) |
| Rather agree | 1975 (17.6%) | 1024 (20.4%) | 153 (24.3%) | 337 (26.8%) | 117 (29.7%) | 84 (24.9%) | 34 (25.0%) | 3724 (19.6%) |
| Completely agree | 8238 (73.6%) | 3479 (69.5%) | 357 (56.8%) | 620 (49.2%) | 199 (50.5%) | 184 (54.6%) | 64 (47.1%) | 13141 (69.3%) |
| **Satisfied with info** |  |  |  |  |  |  |  |  |
| Very satisfied | 475 (4.2%) | 195 (3.9%) | 47 (7.5%) | 120 (9.5%) | 36 (9.1%) | 26 (7.7%) | 11 (8.1%) | 910 (4.8%) |
| Rather satisfied | 1457 (13.0%) | 575 (11.5%) | 103 (16.4%) | 225 (17.9%) | 62 (15.7%) | 53 (15.7%) | 17 (12.5%) | 2492 (13.1%) |
| Neutral | 1842 (16.5%) | 839 (16.8%) | 147 (23.4%) | 226 (18.0%) | 71 (18.0%) | 74 (22.0%) | 46 (33.8%) | 3245 (17.1%) |
| Rather not satisfied | 5114 (45.7%) | 2290 (45.7%) | 242 (38.5%) | 522 (41.5%) | 168 (42.6%) | 143 (42.4%) | 48 (35.3%) | 8527 (45.0%) |
| Very unsatisfied | 2306 (20.6%) | 1109 (22.1%) | 90 (14.3%) | 166 (13.2%) | 57 (14.5%) | 41 (12.2%) | 14 (10.3%) | 3783 (20.0%) |
| **Clarity of official info** |  |  |  |  |  |  |  |  |
| Very clear | 663 (5.9%) | 291 (5.8%) | 64 (10.2%) | 181 (14.4%) | 49 (12.4%) | 36 (10.7%) | 19 (14.0%) | 1303 (6.9%) |
| Rather clear | 1707 (15.2%) | 789 (15.8%) | 111 (17.6%) | 275 (21.8%) | 78 (19.8%) | 89 (26.4%) | 28 (20.6%) | 3077 (16.2%) |
| Neutral | 1551 (13.9%) | 750 (15.0%) | 79 (12.6%) | 175 (13.9%) | 57 (14.5%) | 48 (14.2%) | 23 (16.9%) | 2683 (14.2%) |
| Not clear | 4578 (40.9%) | 2043 (40.8%) | 269 (42.8%) | 450 (35.7%) | 142 (36.0%) | 119 (35.3%) | 47 (34.6%) | 7648 (40.3%) |
| Very unclear | 2695 (24.1%) | 1135 (22.7%) | 106 (16.9%) | 178 (14.1%) | 68 (17.3%) | 45 (13.4%) | 19 (14.0%) | 4246 (22.4%) |
| **Worried w/ PPE** |  |  |  |  |  |  |  |  |
| No | 2007 (17.9%) | 908 (18.1%) | 112 (17.8%) | 182 (14.5%) | 64 (16.2%) | 64 (19.0%) | 30 (22.1%) | 3367 (17.8%) |
| Rather not | 2276 (20.3%) | 1137 (22.7%) | 112 (17.8%) | 296 (23.5%) | 98 (24.9%) | 96 (28.5%) | 28 (20.6%) | 4043 (21.3%) |
| Rather yes | 3373 (30.1%) | 1602 (32.0%) | 239 (38.0%) | 396 (31.5%) | 131 (33.2%) | 106 (31.5%) | 39 (28.7%) | 5886 (31.0%) |
| Yes | 3538 (31.6%) | 1361 (27.2%) | 166 (26.4%) | 385 (30.6%) | 101 (25.6%) | 71 (21.1%) | 39 (28.7%) | 5661 (29.9%) |
| **Worried w/ essential products** |  |  |  |  |  |  |  |  |
| Not worried | 5938 (53.0%) | 2631 (52.5%) | 333 (52.9%) | 662 (52.6%) | 218 (55.3%) | 191 (56.7%) | 81 (59.6%) | 10054 (53.0%) |
| Worried but has access | 2829 (25.3%) | 1166 (23.3%) | 117 (18.6%) | 215 (17.1%) | 49 (12.4%) | 56 (16.6%) | 19 (14.0%) | 4451 (23.5%) |
| Worried and has less access | 2427 (21.7%) | 1211 (24.2%) | 179 (28.5%) | 382 (30.3%) | 127 (32.2%) | 90 (26.7%) | 36 (26.5%) | 4452 (23.5%) |
| **COVID contamination risk** |  |  |  |  |  |  |  |  |
| COVID-negative and no contact with people | 8131 (72.6%) | 3867 (77.2%) | 460 (73.1%) | 975 (77.4%) | 283 (71.8%) | 251 (74.5%) | 104 (76.5%) | 14071 (74.2%) |
| COVID-negative and in contact with people not contaminated | 1635 (14.6%) | 606 (12.1%) | 105 (16.7%) | 152 (12.1%) | 69 (17.5%) | 43 (12.8%) | 16 (11.8%) | 2626 (13.9%) |
| Contaminated OR in contact with contaminated people | 1428 (12.8%) | 535 (10.7%) | 64 (10.2%) | 132 (10.5%) | 42 (10.7%) | 43 (12.8%) | 16 (11.8%) | 2260 (11.9%) |
| **Worry: Financial situation** |  |  |  |  |  |  |  |  |
| Certainly not | 1517 (13.6%) | 781 (15.6%) | 100 (15.9%) | 202 (16.0%) | 74 (18.8%) | 55 (16.3%) | 30 (22.1%) | 2759 (14.6%) |
| Probably not | 3702 (33.1%) | 1744 (34.8%) | 204 (32.4%) | 416 (33.0%) | 126 (32.0%) | 106 (31.5%) | 47 (34.6%) | 6345 (33.5%) |
| Probably | 2966 (26.5%) | 1233 (24.6%) | 168 (26.7%) | 329 (26.1%) | 102 (25.9%) | 78 (23.1%) | 34 (25.0%) | 4910 (25.9%) |
| Very likely | 3009 (26.9%) | 1250 (25.0%) | 157 (25.0%) | 312 (24.8%) | 92 (23.4%) | 98 (29.1%) | 25 (18.4%) | 4943 (26.1%) |
| **Worry: Precar.** |  |  |  |  |  |  |  |  |
| Certainly not | 4157 (37.1%) | 2058 (41.1%) | 240 (38.2%) | 519 (41.2%) | 188 (47.7%) | 149 (44.2%) | 49 (36.0%) | 7360 (38.8%) |
| Probably not | 4871 (43.5%) | 2141 (42.8%) | 271 (43.1%) | 523 (41.5%) | 143 (36.3%) | 131 (38.9%) | 64 (47.1%) | 8144 (43.0%) |
| Probably | 2166 (19.3%) | 809 (16.2%) | 118 (18.8%) | 217 (17.2%) | 63 (16.0%) | 57 (16.9%) | 23 (16.9%) | 3453 (18.2%) |
| **Cope: Words from others** |  |  |  |  |  |  |  |  |
| No | 7337 (65.5%) | 3373 (67.4%) | 439 (69.8%) | 832 (66.1%) | 270 (68.5%) | 231 (68.5%) | 94 (69.1%) | 12576 (66.3%) |
| Yes | 3857 (34.5%) | 1635 (32.6%) | 190 (30.2%) | 427 (33.9%) | 124 (31.5%) | 106 (31.5%) | 42 (30.9%) | 6381 (33.7%) |
| **Cope: Positive beliefs** |  |  |  |  |  |  |  |  |
| No | 5621 (50.2%) | 2628 (52.5%) | 372 (59.1%) | 823 (65.4%) | 251 (63.7%) | 215 (63.8%) | 88 (64.7%) | 9998 (52.7%) |
| Yes | 5573 (49.8%) | 2380 (47.5%) | 257 (40.9%) | 436 (34.6%) | 143 (36.3%) | 122 (36.2%) | 48 (35.3%) | 8959 (47.3%) |
| **Cope: Science** |  |  |  |  |  |  |  |  |
| No | 7774 (69.4%) | 3402 (67.9%) | 423 (67.2%) | 956 (75.9%) | 285 (72.3%) | 232 (68.8%) | 112 (82.4%) | 13184 (69.5%) |
| Yes | 3420 (30.6%) | 1606 (32.1%) | 206 (32.8%) | 303 (24.1%) | 109 (27.7%) | 105 (31.2%) | 24 (17.6%) | 5773 (30.5%) |
| **Cope: Religion** |  |  |  |  |  |  |  |  |
| No | 10390 (92.8%) | 4660 (93.1%) | 576 (91.6%) | 1178 (93.6%) | 360 (91.4%) | 307 (91.1%) | 126 (92.6%) | 17597 (92.8%) |
| Yes | 804 (7.2%) | 348 (6.9%) | 53 (8.4%) | 81 (6.4%) | 34 (8.6%) | 30 (8.9%) | 10 (7.4%) | 1360 (7.2%) |
| **Cope: Resilience** |  |  |  |  |  |  |  |  |
| No | 6246 (55.8%) | 2725 (54.4%) | 302 (48.0%) | 613 (48.7%) | 197 (50.0%) | 158 (46.9%) | 73 (53.7%) | 10314 (54.4%) |
| Yes | 4948 (44.2%) | 2283 (45.6%) | 327 (52.0%) | 646 (51.3%) | 197 (50.0%) | 179 (53.1%) | 63 (46.3%) | 8643 (45.6%) |
| **Cope: Collective** |  |  |  |  |  |  |  |  |
| No | 7517 (67.2%) | 3449 (68.9%) | 429 (68.2%) | 832 (66.1%) | 280 (71.1%) | 217 (64.4%) | 105 (77.2%) | 12829 (67.7%) |
| Yes | 3677 (32.8%) | 1559 (31.1%) | 200 (31.8%) | 427 (33.9%) | 114 (28.9%) | 120 (35.6%) | 31 (22.8%) | 6128 (32.3%) |
| **Cope: Planet** |  |  |  |  |  |  |  |  |
| No | 5335 (47.7%) | 2320 (46.3%) | 294 (46.7%) | 592 (47.0%) | 207 (52.5%) | 170 (50.4%) | 74 (54.4%) | 8992 (47.4%) |
| Yes | 5859 (52.3%) | 2688 (53.7%) | 335 (53.3%) | 667 (53.0%) | 187 (47.5%) | 167 (49.6%) | 62 (45.6%) | 9965 (52.6%) |
| **Cope: Individual** |  |  |  |  |  |  |  |  |
| No | 8358 (74.7%) | 3654 (73.0%) | 469 (74.6%) | 881 (70.0%) | 287 (72.8%) | 235 (69.7%) | 99 (72.8%) | 13983 (73.8%) |
| Yes | 2836 (25.3%) | 1354 (27.0%) | 160 (25.4%) | 378 (30.0%) | 107 (27.2%) | 102 (30.3%) | 37 (27.2%) | 4974 (26.2%) |
| **Cope: Nil** |  |  |  |  |  |  |  |  |
| No | 10565 (94.4%) | 4681 (93.5%) | 580 (92.2%) | 1176 (93.4%) | 365 (92.6%) | 317 (94.1%) | 119 (87.5%) | 17803 (93.9%) |
| Yes | 629 (5.6%) | 327 (6.5%) | 49 (7.8%) | 83 (6.6%) | 29 (7.4%) | 20 (5.9%) | 17 (12.5%) | 1154 (6.1%) |
| **Support: Family** |  |  |  |  |  |  |  |  |
| No | 4456 (39.8%) | 2223 (44.4%) | 292 (46.4%) | 586 (46.5%) | 209 (53.0%) | 173 (51.3%) | 79 (58.1%) | 8018 (42.3%) |
| Yes | 6738 (60.2%) | 2785 (55.6%) | 337 (53.6%) | 673 (53.5%) | 185 (47.0%) | 164 (48.7%) | 57 (41.9%) | 10939 (57.7%) |
| **Support: Friends** |  |  |  |  |  |  |  |  |
| No | 4198 (37.5%) | 2112 (42.2%) | 290 (46.1%) | 527 (41.9%) | 187 (47.5%) | 152 (45.1%) | 73 (53.7%) | 7539 (39.8%) |
| Yes | 6996 (62.5%) | 2896 (57.8%) | 339 (53.9%) | 732 (58.1%) | 207 (52.5%) | 185 (54.9%) | 63 (46.3%) | 11418 (60.2%) |
| **Support: Colleagues** |  |  |  |  |  |  |  |  |
| No | 6838 (61.1%) | 3133 (62.6%) | 429 (68.2%) | 847 (67.3%) | 277 (70.3%) | 242 (71.8%) | 105 (77.2%) | 11871 (62.6%) |
| Yes | 4356 (38.9%) | 1875 (37.4%) | 200 (31.8%) | 412 (32.7%) | 117 (29.7%) | 95 (28.2%) | 31 (22.8%) | 7086 (37.4%) |
| **Support: Neighbors** |  |  |  |  |  |  |  |  |
| No | 9261 (82.7%) | 4091 (81.7%) | 508 (80.8%) | 1000 (79.4%) | 330 (83.8%) | 278 (82.5%) | 122 (89.7%) | 15590 (82.2%) |
| Yes | 1933 (17.3%) | 917 (18.3%) | 121 (19.2%) | 259 (20.6%) | 64 (16.2%) | 59 (17.5%) | 14 (10.3%) | 3367 (17.8%) |
| **Support: Under same roof** |  |  |  |  |  |  |  |  |
| No | 5074 (45.3%) | 2516 (50.2%) | 337 (53.6%) | 631 (50.1%) | 199 (50.5%) | 174 (51.6%) | 89 (65.4%) | 9020 (47.6%) |
| Yes | 6120 (54.7%) | 2492 (49.8%) | 292 (46.4%) | 628 (49.9%) | 195 (49.5%) | 163 (48.4%) | 47 (34.6%) | 9937 (52.4%) |

* Not presented in Table 1 (i.e. social contacts : face to face contacts, telephone calls, text messages and contacts from social networks)

Legend. PPE, Personal Protective Equipment.

**Supplementary Table 2. Effects* of background characteristics on mental well-being.**

| ***Predictor*** | ***lb*** | ***Median*** | ***ub*** |
| --- | --- | --- | --- |
| **Current psych.** | **-3.8198** | **-2.2573** | **-1.3882** |
| **Past psych.** | **-2.7464** | **-1.2697** | **-0.3769** |
| Chronic medical pb. | -1.6109 | -0.6807 | 0.2116 |
| Student | -1.8245 | -0.8775 | 0.2718 |
| Sex: Female | -1.3485 | -0.2920 | 0.4585 |
| Semi-urban | -1.0973 | -0.1268 | 0.5624 |
| Educ: 12 to 14 y/ | -1.0724 | -0.0797 | 0.8945 |
| dis.75 | -1.2719 | -0.2126 | 1.3504 |
| **Age** | **0.4672** | **0.7095** | **1.1437** |
| **Contacts: Phone** | **0.0376** | **0.3644** | **0.6896** |
| Contacts: Text | -0.0426 | 0.3005 | 0.6929 |
| In a relationship | -0.0429 | 0.5977 | 1.4616 |
| Contacts: Face to face | -0.0677 | 0.2122 | 0.5635 |
| Contacts: Soc. network | -0.1519 | 0.1096 | 0.4052 |
| Access to outdoor space | -0.2228 | 0.8144 | 1.7568 |
| Retired | -0.3070 | 0.8983 | 1.9930 |
| Self-employed | -0.4168 | 0.6398 | 2.0167 |
| dis.69 | -0.4207 | 0.2888 | 1.0961 |
| Employee | -0.4440 | 0.2502 | 0.9635 |
| Educ: Bach. to Masters | -0.4480 | 0.2167 | 0.9802 |
| Rural | -0.5549 | 0.2844 | 1.0280 |
| Pet | -0.5672 | 0.1201 | 0.7964 |
| Prev. Lockdown | -0.5708 | 0.2588 | 1.1208 |
| Educ: 14 y/ to Bach. | -0.6825 | 0.0825 | 1.1363 |
| Educ: Masters to PhD | -0.7962 | 0.2290 | 1.5223 |

* Each estimate represents the effect of each predictor over the entire study period (7 weeks of lockdown).

Legend. lb: lower bound of the 95 % confidence interval; ub: upper bound of the 95 % confidence interval. Bach.: Bachelor level. dis.: district.

**Supplementary Table 3. Effects* of COVID-related factors on mental well-being.**

| ***Predictor*** | ***lb*** | ***Median*** | ***ub*** |
| --- | --- | --- | --- |
| **Worry: Precar.** | **-1.2195** | **-0.5652** | **-0.1608** |
| **Worry: Essential products** | **-0.8937** | **-0.3673** | **-0.0392** |
| Worry: PPE | -0.6441 | -0.2786 | 0.0395 |
| Worry: Financial sit. | -0.3653 | -0.0522 | 0.2652 |
| Lockdown w/ family | -1.0266 | -0.2359 | 0.4060 |
| Semi-urban | -1.0973 | -0.1268 | 0.5624 |
| **Satisfied w/ info** | **0.3067** | **0.5975** | **0.9733** |
| **Agree w/ lockdown** | **0.2516** | **0.5975** | **1.0399** |
| Clarity of official info | -0.1192 | 0.1791 | 0.4315 |
| COVID neg. & contact w/ others | -0.1244 | 0.7117 | 1.7705 |
| Access to outdoor space | -0.2228 | 0.8144 | 1.7568 |
| Rural | -0.5549 | 0.2844 | 1.0280 |
| COVID pos. or High risk | -1.0272 | 0.0075 | 1.0200 |

* Each estimate represents the effect of each predictor over the entire study period (7 weeks of lockdown).

Legend. lb: lower bound of the 95 % confidence interval; ub: upper bound of the 95 % confidence interval. PPE: personal protective equipment.

**Supplementary Table 4. Effects* of coping strategies on mental well-being.**

| ***Predictor*** | ***lb*** | ***Median*** | ***ub*** |
| --- | --- | --- | --- |
| Cope: Words from people | -1.0888 | -0.2265 | 0.3966 |
| Cope: Nil | -2.2044 | -0.6303 | 0.9828 |
| **Cope: Resilience** | **0.7464** | **1.3491** | **2.3340** |
| **Cope: Positive beliefs** | **0.4845** | **1.1369** | **1.8262** |
| **Cope: Benefits to indiv.** | **0.1644** | **0.7786** | **1.6484** |
| Cope: Collective | -0.1463 | 0.6103 | 1.2406 |
| Support: Under same roof | -0.1466 | 0.4826 | 1.2172 |
| Support: Colleagues | -0.2464 | 0.4037 | 1.1189 |
| Cope: Benefits to planet | -0.2819 | 0.3687 | 1.0046 |
| Support: Friends | -0.2911 | 0.3053 | 1.0485 |
| Support: Family | -0.3108 | 0.3069 | 1.0001 |
| Support: Neighbors | -0.3368 | 0.6770 | 1.3448 |
| Cope: Science | -0.6824 | 0.0629 | 0.7028 |
| Cope: Religion | -0.7886 | 0.3707 | 1.5175 |

* Each estimate represents the effect of each predictor over the entire study period (7 weeks of lockdown).

Legend. lb: lower bound of the 95 % confidence interval; ub: upper bound of the 95 % confidence interval.

**Supplementary Table 5. Temporal effects* of background characteristics on mental well-being.**

| ***Predictor*** | ***lb*** | ***Median*** | ***ub*** |
| --- | --- | --- | --- |
| **Current psych.** | **-0.6338** | **-0.3659** | **-0.0381** |
| Student | -0.6546 | -0.3095 | 0.0105 |
| Educ: 14 y/ to Bach. | -0.5246 | -0.1773 | 0.0432 |
| Chronic medical pb. | -0.4153 | -0.1871 | 0.0611 |
| Contacts: Face to face | -0.1058 | -0.0163 | 0.0612 |
| Contacts: Soc. network | -0.0870 | -0.0147 | 0.0639 |
| Past psych. | -0.4455 | -0.1909 | 0.1086 |
| Educ: Bach. to Masters | -0.2720 | -0.0644 | 0.1100 |
| Employee | -0.2586 | -0.0568 | 0.1216 |
| dis.69 | -0.2852 | -0.0469 | 0.1668 |
| dis.75 | -0.7415 | -0.1822 | 0.2248 |
| Educ: Masters to PhD | -0.5334 | -0.0611 | 0.2431 |
| Sex: Female | -0.1855 | -0.0044 | 0.2960 |
| Age | -0.0009 | 0.0032 | 0.0081 |
| Contacts: Phone | -0.0541 | 0.0325 | 0.1327 |
| Contacts: Text | -0.0706 | 0.0056 | 0.0970 |
| Semi-urban | -0.1500 | 0.0485 | 0.3206 |
| Access to outdoor space | -0.1540 | 0.0310 | 0.2886 |
| Pet | -0.1790 | 0.0030 | 0.2123 |
| Prev. Lockdown | -0.1996 | 0.0292 | 0.2710 |
| In a relationship | -0.2170 | 0.0013 | 0.1688 |
| Rural | -0.2353 | 0.0022 | 0.2273 |
| Educ: 12 to 14 y/ | -0.2647 | 0.0145 | 0.3034 |
| Self-employed | -0.3658 | 0.0119 | 0.3457 |
| Retired | -0.4124 | 0.1263 | 0.5097 |

* Each estimate represents the effect of each predictor for 10 days of lockdown.

Legend. lb: lower bound of the 95 % confidence interval; ub: upper bound of the 95 % confidence interval. Bach.: Bachelor level. dis.: district.

**Supplementary Table 6. Temporal effects* of COVID-related factors on mental well-being.**

| ***Predictor*** | ***lb*** | ***Median*** | ***ub*** |
| --- | --- | --- | --- |
| **Time** | **-0.6195** | **-0.2989** | **-0.1507** |
| **Worry: PPE** | **-0.1791** | **-0.0894** | **-0.0054** |
| Agree w/ lockdown | -0.0827 | -0.0100 | 0.0483 |
| Worry: Financial sit. | -0.1272 | -0.0345 | 0.0496 |
| Worry: Precar. | -0.2159 | -0.0906 | 0.0642 |
| Lockdown w/ family | -0.2536 | -0.0660 | 0.1388 |
| Worry: Essential products | -0.0999 | -0.0071 | 0.1610 |
| COVID pos. or High risk | -0.3539 | -0.0627 | 0.2070 |
| Satisfied w/ info | -0.0263 | 0.0459 | 0.1184 |
| Clarity of official info | -0.0547 | 0.0099 | 0.0825 |
| Semi-urban | -0.1500 | 0.0485 | 0.3206 |
| Access to outdoor space | -0.1540 | 0.0310 | 0.2886 |
| Rural | -0.2353 | 0.0022 | 0.2273 |
| COVID neg. & contact w/ others | -0.2921 | 0.0322 | 0.2910 |

* Each estimate represents the effect of each predictor for 10 days of lockdown.

Legend. lb: lower bound of the 95 % confidence interval; ub: upper bound of the 95 % confidence interval. PPE: personal protective equipment.

**Supplementary Table 7. Temporal effects* of coping strategies on mental well-being.**

| ***Predictor*** | ***lb*** | ***Median*** | ***ub*** |
| --- | --- | --- | --- |
| Support: Family | -0.2824 | -0.0414 | 0.1265 |
| Support: Friends | -0.2096 | -0.0200 | 0.1529 |
| Cope: Nil | -0.5137 | -0.1064 | 0.4040 |
| **Support: Neighbors** | **0.0446** | **0.2924** | **0.6234** |
| **Cope: Positive beliefs** | **0.0420** | **0.2417** | **0.4403** |
| **Cope: Collective** | **0.0385** | **0.2298** | **0.4566** |
| Cope: Resilience | -0.0957 | 0.1433 | 0.3212 |
| Cope: Benefits to planet | -0.1173 | 0.0633 | 0.2676 |
| Cope: Benefits to indiv. | -0.1508 | 0.0880 | 0.2667 |
| Support: Under same roof | -0.1668 | 0.0435 | 0.2515 |
| Support: Colleagues | -0.1694 | 0.0455 | 0.2316 |
| Cope: Words from people | -0.1931 | 0.0038 | 0.2996 |
| Cope: Science | -0.2118 | 0.0180 | 0.2473 |
| Cope: Religion | -0.3007 | 0.0936 | 0.4377 |

* Each estimate represents the effect of each predictor for 10 days of lockdown.

Legend. lb: lower bound of the 95 % confidence interval; ub: upper bound of the 95 % confidence interval.
